# Graduate Students Take to the Field in K–12 Education

**DOI:** 10.1371/journal.pbio.0050162

**Published:** 2007-06-12

**Authors:** Betsy J Mitchell, Rosemary G Gillespie

## Abstract

By providing fellowships to graduate students, who then design and teach science activities, the National Science Foundation offers a way to bring the resources and expertise of universities to school children.

Children are born curious, and nature is one of the most compelling targets for their curiosity. Unfortunately, as the world becomes more urbanized, interactions between children and the natural world are becoming all too rare. Children today often express fear, apathy, or disdain toward nature, and some don't even recognize plants as living things. In many cases a child's parents or siblings are equally alienated from natural areas, and so cannot serve as role models to help preserve or mentor the child's innate curiosity about nature. While this problem is widespread, it is especially troubling in California where, with a rapidly expanding human population, we face increasing degradation and loss of wild lands, with native species threatened by extinction through habitat modification and invasion of exotic species. The irony here is that California is one of the world's biodiversity hotspots, a fact not recognized by many of its citizens.

Biology classes are one place where children can be introduced to nature, where they can gain an understanding and appreciation for the diversity of life, its importance and its wonder. Moreover, activities that engage children in their own explorations of natural history can energize their interest in school, in science, and in important environmental issues. Unfortunately, there are a number of barriers that not only prevent teachers from teaching about the diversity of life but also prevent students from exploring it. One barrier is that many teachers do not have a strong background in natural science. Another is that they often have poor access to natural areas for study, either because there are none nearby or because the teachers lack the funds to reach such sites. In addition, with current emphasis on academic improvement (based on literacy, math, and standardized tests) and large class loads, there is often little time for field trips or hands-on activities. Universities could help by providing their expertise. But, again, heavy demands on professors' time, little experience communicating with non-university audiences, and the lack of incentives to reach out to school children have limited the exchange between university scientists and Kindergarten through 12th grade (K–12) communities.

**Figure:**
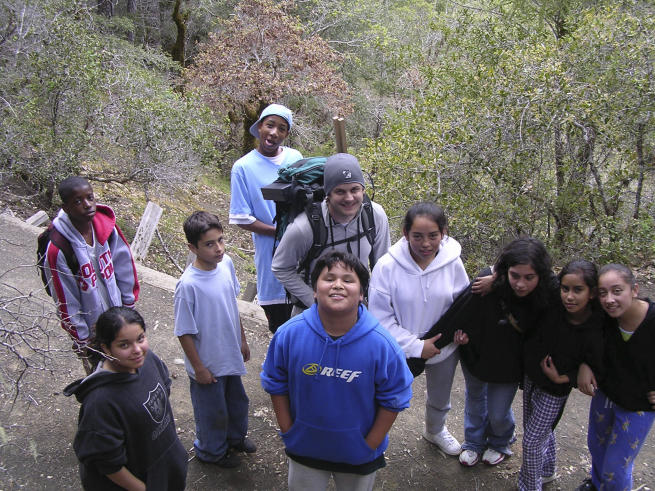
Brian Kraatz, a graduate student at the University of California Museum of Paleontology, introduces Adams Middle School students to the flora and fauna of the Heath and Marjorie Angelo Coast Range Reserve in Mendocino County.

The National Science Foundation is addressing this problem with its Graduate Teaching Fellows in K–12 Education Program (GK–12). By providing fellowships to graduate students who can then design and teach science activities in K–12 schools, this program offers a way to bring the resources and expertise of universities to the K–12 community. It also offers graduate students an unparalleled opportunity to acquire and practice communication skills that will enhance their professional and scientific careers. Our project at the University of California Berkeley Natural History Museums, “Exploring California Biodiversity” (http://gk12calbio.berkeley.edu), is one of many GK–12 projects at universities across the country. We have worked with over 700 students in middle and high schools across the San Francisco Bay Area over the past four years. A major goal of our project is to inspire in urban children an appreciation for the amazing diversity of life on earth, an understanding of processes that affect biodiversity, and recognition that biodiversity is not confined to the rainforests of exotic places, but can be found within their own school yards. Graduate fellows work with teachers and their students using the facilities and resources of the University's museums and field stations to bring examples of biodiversity to the classroom and to take students into the field. Classes also explore biodiversity in their schoolyards with a variety of activities, including mapping habitats, collecting and identifying insects and plants, and building a mini-museum.

Working with graduate fellows gives K–12 classes exposure to the excitement of discovery, where they learn about science as it is happening, and use multiple sensory modalities—touch, smell, feel—in addition to simply looking and listening. The impact of the program can be seen directly in the rise in class attendance figures at these schools, and teachers report that many of their students express an increased interest in science and a more positive attitude toward school. Perhaps the most gratifying indicator of our success is seeing so many of the students who started the year running away from insects and spiders end the year easily holding them in their hands. The graduate students in the program learn to communicate in different environments, which in turn has often provided them with renewed understanding and excitement for their own research. Teachers enjoy the new ideas and knowledge the fellows bring to their classes, and the added help enables them to include more hands-on activities in their curricula.

Programs like ours that link university scientists with K–12 teachers and their students in the hands-on study of organisms in their natural habitats seek to foster a generation of young adults that is scientifically literate and understands the value of biodiversity. If we are successful, these students will become responsible stewards of the natural world and make wise decisions about environmental challenges facing our society. Given the current status of the global environment, building on these efforts has never been more critical.

## Abbreviations

GK–12: Graduate Teaching Fellows in K–12 Education Program

